# Chimpanzee Vocal Signaling Points to a Multimodal Origin of Human Language

**DOI:** 10.1371/journal.pone.0018852

**Published:** 2011-04-20

**Authors:** Jared P. Taglialatela, Jamie L. Russell, Jennifer A. Schaeffer, William D. Hopkins

**Affiliations:** 1 Department of Biology and Physics, Kennesaw State University, Kennesaw, Georgia, United States of America; 2 Division of Developmental and Cognitive Neuroscience, Yerkes National Primate Research Center, Atlanta, Georgia, United States of America; 3 Department of Psychology, Agnes Scott College, Decatur, Georgia, United States of America; University of Rennes 1, France

## Abstract

The evolutionary origin of human language and its neurobiological foundations has long been the object of intense scientific debate. Although a number of theories have been proposed, one particularly contentious model suggests that human language evolved from a manual gestural communication system in a common ape-human ancestor. Consistent with a gestural origins theory are data indicating that chimpanzees intentionally and referentially communicate via manual gestures, and the production of manual gestures, in conjunction with vocalizations, activates the chimpanzee Broca’s area homologue – a region in the human brain that is critical for the planning and execution of language. However, it is not known if this activity observed in the chimpanzee Broca’s area is the result of the chimpanzees producing manual communicative gestures, communicative sounds, or both. This information is critical for evaluating the theory that human language evolved from a strictly manual gestural system. To this end, we used positron emission tomography (PET) to examine the neural metabolic activity in the chimpanzee brain. We collected PET data in 4 subjects, all of whom produced manual communicative gestures. However, 2 of these subjects also produced so-called attention-getting vocalizations directed towards a human experimenter. Interestingly, only the two subjects that produced these attention-getting sounds showed greater mean metabolic activity in the Broca’s area homologue as compared to a baseline scan. The two subjects that did not produce attention-getting sounds did not. These data contradict an exclusive “gestural origins” theory for they suggest that it is vocal signaling that selectively activates the Broca’s area homologue in chimpanzees. In other words, the activity observed in the Broca’s area homologue reflects the production of vocal signals by the chimpanzees, suggesting thast this critical human language region was involved in vocal signaling in the common ancestor of both modern humans and chimpanzees.

## Introduction

The study of communicative behavior in extant nonhuman primates is critical for understanding the evolutionary origins of human language and the biological substrates that support these competencies. However, data from the species with the closest phylogenetic relation to humans, chimpanzees, are relatively scarce. Notwithstanding, theories concerning the origin of human language and the neural substrates that support this capacity have proposed that (in some form or another), human spoken language is fundamentally different from other animal vocal communication systems. Specifically, much of the data available on nonhuman primates suggest that, in contrast to humans, vocal production is relatively fixed in both form and usage [1], [2], (but see [3]). In contrast, relatively recent data indicate that the manual communicative gestures of apes are learned, used flexibly, and are intentionally produced [4], [5], [6], [7], [8], [9]. For example, chimpanzees, and other great apes, produce manual communicative gestures only when a human is present and visually oriented towards them [4], [5], [6], [8], [9], [10], [11]. In such situations, chimpanzees alternate their gaze between a referent (food) and a social agent while gesturing [12] and ‘repair’ these communicative attempts when they have failed [7].

Therefore, it is somewhat difficult to reconcile that in the vocal domain, chimpanzees (and other apes), use a relatively limited number of sounds in fixed contexts, but in the gestural domain, chimpanzees seem to be able to use their signals flexibly and with specific intent. This dichotomy has led some researchers to conclude that human language must have evolved from a manual gestural communicative system [13]. However, others have noted that although nonhuman primate vocal *production* is relatively fixed, *comprehension* is quite flexible, suggesting continuity among the vocalizations of nonhuman primates and human spoken language [1], [2]. In fact, recent data suggest that nonhuman primate vocal production, *as well as* usage and comprehension, demonstrates a considerable degree of control and flexibility [3]. Given the available data, both a gestural origins theory and vocal origins theory seem plausible. However, for those interested in the evolutionary origins of human language, and specifically those characteristics and competencies that likely evolved following the split between chimpanzees and humans, it is critical to distinguish between these two theories.

Specifically, a gestural origins theory proposes that chimpanzee gestures, in contrast to vocalizations, are produced flexibly and with specific intent. However, in captivity, chimpanzees also produce vocalizations (often in conjunction with their manual communicative gestures) that are directed at human experimenters [14], [15]. Typically, chimpanzees will use a specific type of call – so-called “attention-getting” vocalizations – to capture the attention of an otherwise inattentive human [16], as well as a number of other acoustic signals including hand clapping, banging, etc. In these situations, chimpanzees will typically employ the acoustic signal first to capture the attention of the human, and then produce a visual signal to make a request, (e.g. a manual gesture to request a piece of food that the experimenter has).

Previous data indicate that the production of these attention-getting calls in conjunction with manual gestures selectively activates the Broca’s area homologue in chimpanzees [17]. Broca’s area, a region of the cerebral cortex located in the left inferior frontal gyrus (IFG) of the human brain, is critical for the planning and execution of language. Since its first identification, this region has received a great deal of scientific attention. However, modern theories concerning the neural correlates of language in the human brain have moved beyond a classical modular approach to language processing. Modern neuroimaging data now point to a distributed network of both cortical and subcortical regions in both hemispheres of the human brain that are responsible for linguistic competency. Notwithstanding, Broca’s area presents a potentially fruitful starting point for examining the evolutionary origins of human language given its critical role in language processing and production. Comparatively, previous data indicate that chimpanzees too have a region of the left inferior frontal gyrus that is anatomically homologous to the human Broca’s area [18], and recent cytoarchitectonic data confirm this anatomical location contains Brodmann’s area 44 and 45 cells [19]. Although functional imaging confirmed that this region is selectively activated during the production of communicative gestures and vocal signals [17], it is not clear if the activity in the left IFG previously observed was the result of the chimpanzees producing manual communicative gestures, communicative sounds, or both. However, these data are vital for evaluating theories that propose a gestural origin of human language. Specifically, if this region of the left IFG is only involved in the production of manual communicative gestures (and not vocal signaling) in chimpanzees, then one can conclude that this critical language region became involved in communicative vocal signaling following the split between humans and chimpanzees some 5 million years ago. However, if the Broca’s area homologue *was* involved in both the production of gestures and vocal signals prior to the split between modern humans and chimpanzees, then a strictly gestural origin for human language would not be supported.

To this end, we conducted a second study in which we again used positron emission tomography (PET) to examine the neural metabolic activity in the chimpanzee brain. We collected PET data in 4 subjects, all of who produced manual communicative gestures directed towards a human experimenter. However, 2 of these subjects also produced attention-getting vocalizations [16] directed towards a human experimenter in conjunction with their manual gestures. (One of these subjects (S2) participated in the previous PET study described above [17]). We hypothesized that if the region of the chimpanzee IFG identified previously is involved in the production of attention-getting vocalizations, those subjects that produced calls in conjunction with manual communicative gestures would show greater metabolic activity in the Broca’s area homologue than those chimpanzees that produced manual communicative gestures only.

## Methods

### Ethics Statement

All aspects of this study were conducted in accordance with ethical guidelines associated with the care and use of nonhuman primates and with the approval of the Emory University Institutional Animal Care and Use Committee (046-2003Y). Although the functional imaging techniques used in this study are noninvasive, the chimpanzees were anesthetized for imaging. In order to minimize potential stress and discomfort associated with the administration of anesthetic, chimpanzees were trained using positive reinforcement to voluntarily present for an intramuscular injection. While under anesthesia, the chimpanzees were continuously monitored by a veterinarian.

### Subjects

Subjects were four captive-born chimpanzees, *(Pan troglodytes)* including two males and two females between the ages of 14 and 31 years. All four subjects were born in captivity and reared by their chimpanzee mother or in a nursery environment at the Yerkes National Primate Research Center (YNPRC) [Bard 1996]. All four subjects live in small social groups (N = 2–12).

To compare neural metabolic activity between those subjects that produced gestures only with those that produced gestures and attention-getting sounds, two of the subjects (1 male, 1 female) were selected as subject given that they had not previously been observed to produce attention-getting sounds (AG-) [16]. The other two subjects (1 male, 1 female) were selected as those that frequently produce attention-getting sounds towards humans (AG+).

### Behavioral tasks

For the communication production task (COM), each subject was separated from their social group, but remained in their home enclosure, and consumed the ^18^F-FDG. A human experimenter would then approach the subject and place a cache of food (1 quart plastic container containing 20–30 small frozen cubes of approximately 2 fluid ounces of sugar-free flavored drink mixture) just outside the subject’s home enclosure at a distance of less than 1 meter, but beyond the subject’s reach. Previous research in our lab has demonstrated that the chimpanzees are likely to produce both manual gestures and vocalizations in these contexts [5], [8], [16]. The human experimenter would remain seated in front of the subject’s enclosure for 2 minutes. The experimenter would verbally acknowledge the subject’s communicative signals, but would not give any of the frozen drink cubes to the subject. At the end of the two minute block, the experimenter would respond to the subject’s next communicative signal by offering a small frozen drink cube to the subject. The human experimenter would then leave the area, taking the container of frozen drink cubes with them. After a two minute interval, the experimenter would return with the container of frozen drink cubes, once again placing them in front of the subject’s enclosure. This procedure was repeated for the duration of the uptake period (40 minutes).

For the baseline resting state condition (RES), subjects were not required to participate in any specific task. They simply remained in their home enclosure for the duration of the uptake period (40 minutes). As in the COM task, each subject was separated from their social group, but remained in their home enclosure, and consumed the ligand. The human experimenter would then sit down at a distance from the subject’s home enclosure. The experimenter would then observe a two minute interval. After the two minutes had expired, the human experimenter would offer a small frozen drink cube to the subject as had been done in the COM task. This procedure was included to serve as a comparison with the COM task. Compared with the COM task, the experimenter positioned themselves farther from the subject, and the cache of food was not visible to the subjects except when they were offered a frozen drink cube. This was done so as to reduce the likelihood that the subject would communicate with the experimenter in the RES condition.

For both the COM and RES conditions, subjects were housed in their home enclosures for the duration of the uptake period. Although physically separated from their social group, the subjects were able to hear conspecifics. This was done to minimize any stress that would have been associated with placing the chimpanzees in an unfamiliar environment, while simultaneously attempting to preserve the authenticity of the communicative interaction for the COM condition. With the exception of the limited speech produced by the experimenter, their own vocalizations, background noises (e.g. building mechanical equipment), and the rare occurrence of a conspecific vocalization, subjects in both conditions had limited auditory input.

Prior to scanning, chimpanzee subjects had been trained using positive reinforcement techniques to present for an injection. Following the behavioral tasks, subjects voluntarily presented for an intramuscular injection of an anesthetic agent and were transported to the PET imaging facility.

### PET procedures

Subjects were administered ^18^F-fluorodeoxyglucose (^18^F-FDG) at a dose of 20 mCi. FDG was selected as the ligand because of its relatively long uptake period (∼80 minutes) and long half-life (approximately 110 minutes). Thus, just as we and other investigators have done previously, we capitalized on these features of ^18^F-FDG because they allowed for prolonged behavioral testing during the uptake period and a relatively long time frame to capture neural activity trapped in the cells between the termination of uptake and the interval of time needed to transport and scan the chimpanzees. Previous studies have used nearly identical procedures to scan other non-human primate species and have revealed significant and consistent patterns of PET activation [17], [20], [21], [22], [23].

Chimpanzees consumed. 24 ml of ^18^F-FDG that was diluted in approximately 100 ml of a sugar free flavored drink mixture. The subjects then participated in the behavioral task for 40 minutes. Following the 40 minute uptake period, chimpanzees were asked to voluntarily present for an intramuscular injection of Telazol (4 mg/kg). Once anesthetized, chimpanzees were transported to the PET imaging facility. For the duration of the PET scan, chimpanzees remained anesthetized with Propofol administered intravenously and diluted in lactated ringers at a dose of ∼10 mg/kg/hr. After completing PET procedures, the subjects were returned to the YNPRC and temporarily housed in a single cage for approximately 18 h to allow the effects of the anesthesia to wear off and radioactivity to decay. Subjects were then returned to their home cages with their social group.

The PET images were acquired on a High Resolution Research Tomograph (CPS HRRT; CTI/Siemens, Inc.) approximately 1 hour and 35 minutes following ingestion of the ^18^F-FDG. Recall that 40 minutes of this time period constituted the uptake period; thus, the remaining 55 minutes constituted the time between the injection of anesthesia, transport to and from the PET imaging and the PET scan duration (approximately 30 minutes). Scan procedures were identical for all subjects. Chimpanzees fasted for approximately 5 hours prior to ^18^F-FDG administration, and were rewarded with only minimal amounts of frozen sugar free flavored drink cubes during the uptake period. Subjects were placed in the supine position inside the scanner. Six minute transmission scans were followed by 20 minute emission scans. Scan parameters were identical for all subjects: Axial FOV = 24 cm; Transverse FOV = 31.2 cm; Slice thickness = 1.21875 mm. Transaxial Spatial Resolution FWHM is 2.4 mm at the center and 2.8 mm 10 cm from the center. Following scanning, a post reconstruction 2 mm smooth was applied to the images.

### MRI procedures

Magnetic resonance images (MRI) were collected from each subject using a 3.0 Tesla scanner (Siemens Trio) at the Yerkes National Primate Research Center (YNPRC). T1-weighted images were collected using a 3D gradient echo sequence (pulse repetition = 2300 ms, echo time = 4.4 ms, number of signals averaged = 3, matrix size = 320×320). The archived MRI data were transferred to an Apple MacBook Pro running Analyze 9.0 (Mayo Clinic) software for post-image processing. MRI scans were then aligned in the axial plane and virtually cut into 1 mm slices using Analyze 9.0.

### Image Processing

The individual PET images were spatially aligned to their respective MR images using 3D voxel registration with a linear transformation (Analyze 9.0, Mayo Clinic). Once aligned, each subject’s MRI was used to outline the brain on the PET image in each and every slice in the axial plane. An average PET activation was then calculated based on the registered activity within these slices. Once the mean activation for the whole brain had been computed, each voxel within that entire volume was divided by the mean activation in order to obtain a standardized PET image.

**Figure 1:**
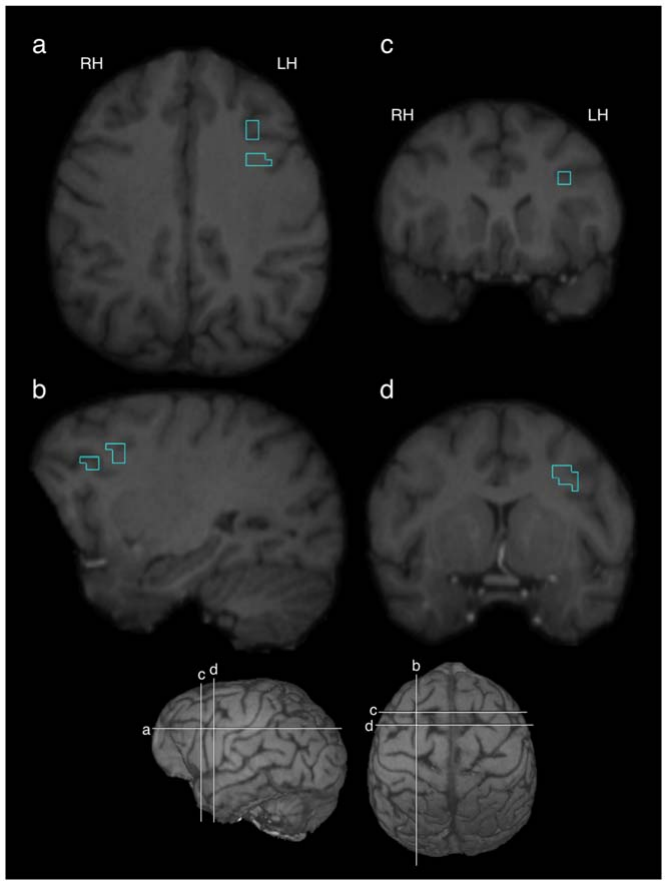
Significant cluster of activation identified previously using PET [17] overlaid on a representative chimpanzee brain. Traces represent boundaries of the region in the three orthogonal planes: transverse (a), sagittal (b), and coronal (c & d). Bottom panel indicates locations of 4 images (a, b, c, & d) on a representative 3-D rendered chimpanzee brain.

The IFG cluster identified previously [17] was then spatially aligned to the each individual subject’s MRI using 3D voxel registration with a linear transformation (Analyze 9.0, Mayo Clinic). Figure 1 displays this region overlaid on the MR image of a representative chimpanzee brain. The mean activation within this region was then calculated by overlaying this previously identified cluster onto the standardized COM and RES volumes for each subject. Difference volumes were then calculated by subtracting each subject’s standardized RES volume from their standardized COM volume.

## Results

**Figure 2:**
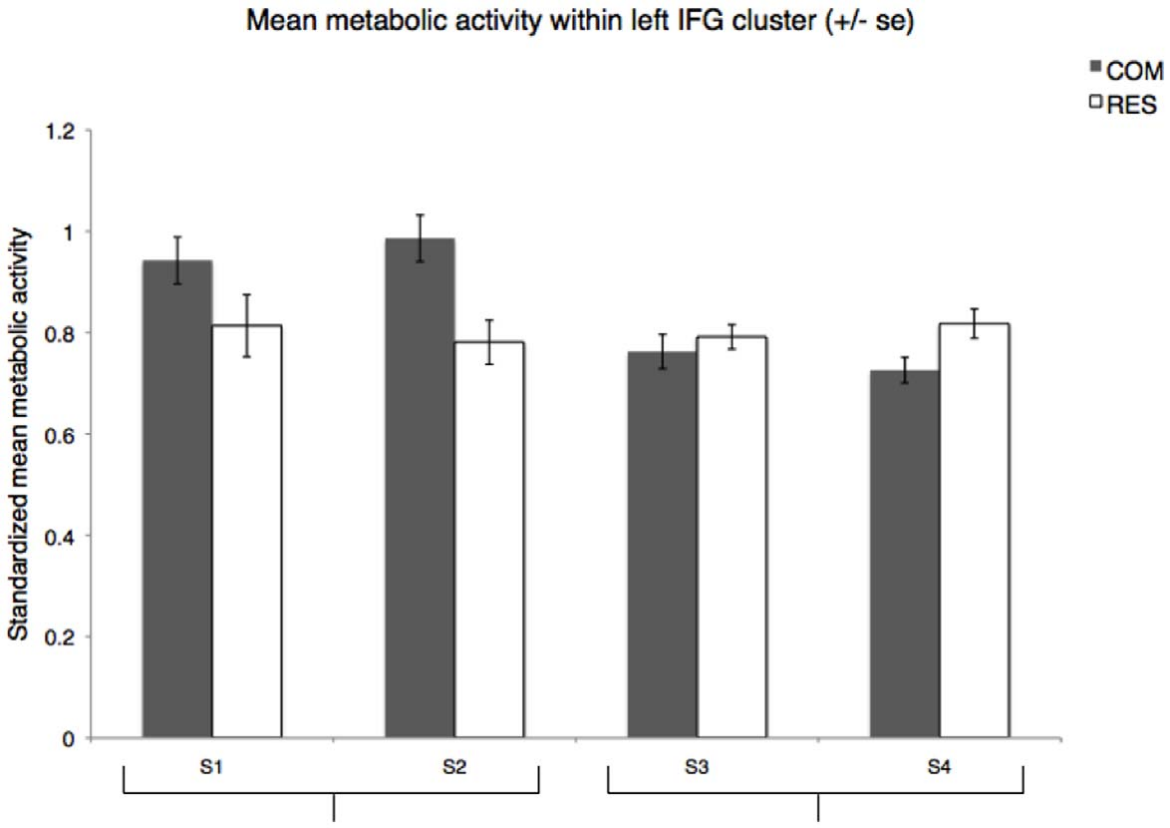
Individual standardized mean metabolic activity for COM and RES conditions. Paired voxel-wise comparisons of the mean metabolic activity in the COM and RES conditions indicated significantly greater activation in the previously identified cluster during COM vs. RES for those subjects that produced AG sounds (AG+) [S1 and S2; t(33) = 5.70, p<001, t(34) = 9.35, p<001, respectively] but not for those that did not produce AG sounds (AG-) [S3 and S4; t(34) = −1.96, p = 06, t(37) = −5.81, p<001, respectively]. Note that for S4 the metabolic activity was actually significantly greater in the RES condition as compared to the COM.

**Figure 3:**
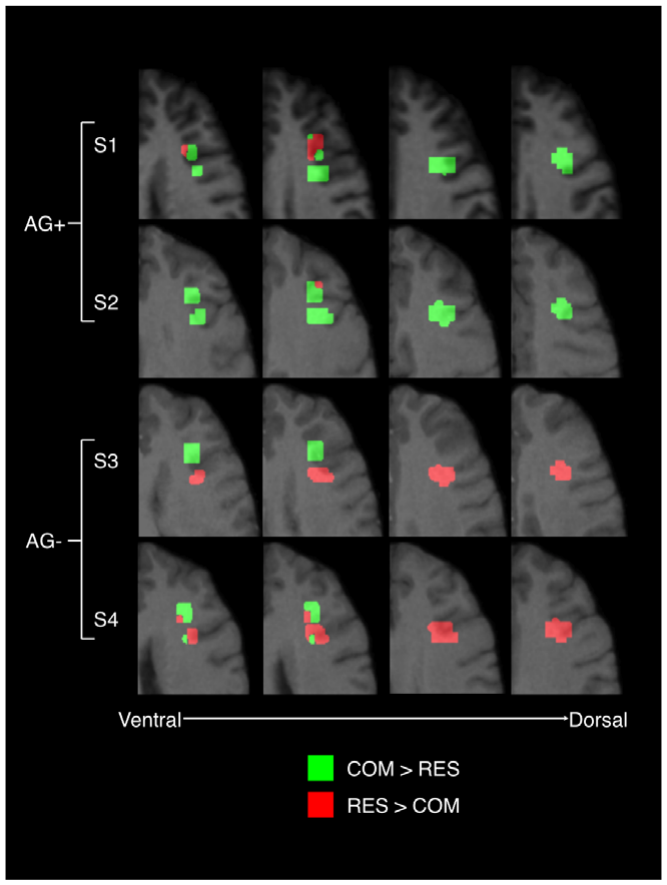
Individual PET data for all 4 subjects. Individual difference PET volumes (COM - RES) for subject 1 (S1), subject 2 (S2) - i.e. those subjects that produced AG vocalizations (AG+), and subject 3 (S3) and subject 4 (S4) - i.e. those subjects that did not produce AG sounds (AG-), overlaid on each subject’s individual MR brain image. Voxels in green indicate positive differences (i.e. metabolic activity is greater in COM vs. RES), whereas voxels in red indicate negative differences (i.e. metabolic activity is greater in RES vs. COM). Difference values of “0” as well as activation outside the region of interest are masked. Images are in transverse plane in serial 2 mm slices from ventral to dorsal as indicated in the figure.

Table 1 depicts the manual gestures, and attention-getting sounds produced by each of the four subjects during both the COM and RES behavioral tasks. Paired comparisons of the mean metabolic activity at each voxel within the previously identified left IFG cluster indicated that the two subjects (one male, one female) that produced communicative vocal signals in conjunction with their manual gestures (AG+) showed significantly greater activity in the COM condition when compared to RES [S1 and S2; t(33) = 5.70, p<001, t(34) = 9.35, p<001, respectively]. However, the two subjects that did not produce attention-getting sounds did not show greater mean metabolic activity in the IFG in the COM condition as compared to the baseline RES condition [S3 and S4; t(34) = −1.96, p = 06, t(37) = −5.81, p<001, respectively] (Figure 2). Figure 3 depicts the difference volumes (COM – RES) for each of the four subjects.

**Table 1:** Attention-getting (AG) calls and manual communicative gestures produced during the uptake period in the COM and RES conditions.

Subject	# AG Calls		# L Gestures		# R Gestures		# Bimanual Gestures	
	COM	RES	COM	RES	COM	RES	COM	RES
Artemus (S1)	82	0	18	0	26	0	15	0
Dara (S2)	89	0	0	0	2	2	0	0
Drew (S3)	0	0	101	3	57	0	1	0
Lena (S4)	0	0	3	0	54	0	1	0

Attention-getting calls included “raspberries,” “kisses,” and “extended grunts” and occurred exclusively in the context of requesting food or attention from a human experimenter [16].

## Discussion

These results indicate that vocal signaling in conjunction with manual communicative gestures selectively activate the Broca’s area homologue in chimpanzees. These data are significant because they suggest that Broca’s area, a cortical region of the human brain that is critical for the production of human language, was involved in the production of communicative oro-facial/vocal signaling in the common ancestor of both humans and chimpanzees. This finding contradicts an exclusive “gestural origins” theory for human language, and points to a multimodal origin of human language where both manual communicative gestures and vocal signals were commonly controlled and coevolved in a common hominid ancestor.

For this study, our aim was to determine if there were differences in the neural correlates associated with the production of attention-getting calls and the neural correlates of manual communicative gestures - both human-directed communicative behaviors produced by individuals in our colony of chimpanzees. To this end, we included two subjects in this study that reliably produce attention-getting calls (AG+), and two that had never been observed to produce these types of calls [16]. As indicated in Table 1, both AG+ subjects produced attention-getting calls during the COM condition, but not during the RES condition. In addition, both AG- subjects did not produce attention-getting calls in either the COM or RES conditions, but did produce a relatively large number of manual communicative gestures (S3 = 159 total gestures; S4 = 58 total gestures) in the COM condition.

It is important to note, that the behaviors produced by all four of the chimpanzee subjects in both the COM and RES conditions were, more or less, self-paced. Therefore, the number and type of signals produced were not directly under the control of the experimenter during the uptake period. Our rationale for this procedure was to create a relatively authentic communicative interaction during the COM condition thereby enabling us to isolate neuronal metabolic activity related to the production of human-directed communicative signals in the oro-facial and/or manual domain. As indicated in Table 2, however, subjects also produced other vocalizations presumably directed to nearby conspecifics and/or the experimenter. These included food-associated calls (e.g. barks and grunts) as well as pant-hoots [24].

**Table 2:** Non-attention-getting calls produced during the uptake period in the COM and RES conditions.

Subject	# PH		# B/G		# GR		# S/W	
	COM	RES	COM	RES	COM	RES	COM	RES
Artemus (S1)	0	0	84	63	5	675	1	0
Dara (S2)	0	0	0	0	0	0	11	1
Drew (S3)	5	1	239	52	0	0	0	1
Lena (S4)	0	3	0	0	0	0	0	0

These sounds were characterized by the fact that they did not occur within the context of requesting food from a human experimenter, and included pant hoots, barks/grunts, grooming vocalizations, whimpers and screams. Pant-hoots (PH) are voiced on both inhalation and exhalation, may incorporate a series of “hoo” sounds escalating to a climactic scream or piercing “ahh” vocalization, and seem to be directed to distant recipients. Barks and grunts (B/G) are relatively short vocalizations that can be tonal (barks) or noisy (grunts), and are often produced in a series. These calls are associated with the anticipation of eating or receiving food, or other positive experiences, as well as during introductions/reunions with social partners.

Grooming calls (GR) are unvoiced sounds that include teeth chomping/clacking, and occur during grooming bouts with another individual or during autogrooming. Screams (S) are relatively loud, high-pitched, voiced shrieks and at its most intense can be raspy or even hoarse sounding. Screams usually occur in contexts of fear, submission, or distress. Whimpers (W) are similar to modulated, high-pitched ‘hoo’ sounds or crying and often progresses into screams. Whimpering occurs in chimpanzees of all ages during distress or fear and by infants when being weaned.

The production of these “non-attention-getting” calls did vary to some extent among the four subjects. Specifically, S1 and S3 both produced barks and grunts in both the COM and RES condition. Whereas S1 produced a similar number of these calls in the both conditions, S3 produced many more barks and grunts in the COM conditioned compared with the RES. Although the conclusions that can be drawn from the behavior of one individual are limited, it is interesting to note that although S3 produced many more barks and grunts in the COM condition compared with the RES condition, the production of these calls did not result in increased neuronal metabolic activity in the left IFG as observed for the AG+ subjects. Therefore, the increased left IFG activity in the COM condition compared to RES reported here cannot be simply attributed to the production of all calls, but specifically to attention-getting sounds [16]. In addition, S1 produced many more grooming sounds in RES compared to COM. As described above, during the RES condition the subjects were free to behave as they wished. During the RES condition, S1 chose to groom himself – thus, the relatively large number of calls associated with autogrooming. Despite the relatively large number of grooming sounds produced in the RES condition (see Table 2), significantly greater activity was nonetheless observed in the left IFG in the COM condition as compared to RES. Again, it appears that the left IFG activity can be attributed specifically to the production of attention-getting calls, and not simply call production in general.

We have previously shown that there are differences in the way different functional classes of vocalizations, are processed in the chimpanzee brain [23]. Specifically, previous data indicate that *broadcast* vocalizations (relatively stereotyped, high amplitude calls) and *proximal* vocalizations (relatively low intensity signals produced in close spatial proximity of conspecifics and used in a variety of communicative and social contexts [16], [25], [26], [27]) are processed differently in the chimpanzee brain [23]. Therefore, it is possible that the *production* of different functional classes of sounds may similarly involve the recruitment of neuronal populations in different cortical regions. However, although attention-getting calls are certainly considered proximal calls, barks and grunts are as well. Therefore, the proximal/broadcast distinction cannot completely account for the differences in neuronal metabolic activity in the COM and RES conditions observed in our subjects (see Table 2).

However, one characteristic of the attention-getting calls used by the chimpanzees in our colony is that they have a clear recipient (i.e. a specific individual that the signaler is attempting to communicate with). It is possible that this distinguishes attention-getting calls from at least some of the other types of calls produced by our subjects, and therefore is reflected in the significant increase in activity in the left IFG in the COM compared to RES condition. Indeed Ghazanfar et al., [28] have proposed this directed/non-directed distinction may explain different patterns of responses in the auditory cortex of macaque monkeys following the presentation of two different types of proximal vocalizations (coos and grunts). It is possible that directed calls, and the contexts in which they are produced, provide a more appropriate setting for comparisons with human language than non-directed calls because the fundamental component of human speech is conversation [29]. Dunbar [30] has proposed that conversation may have played a significant role in the evolution of spoken language.

Consistent with this idea, researchers have examined vocal exchanges between familiar individuals in close spatial proximity of one another in a number of nonhuman primate species [29], [31], [32], [33], [34], [35], [36], [37], [38]. The results of these studies suggest that this type of vocal exchange may share some characteristics with human conversation. For example, female squirrel monkeys preferentially call in response to the vocalizations of familiar group members compared to those produced by unknown individuals [35], [39], exchange calls preferentially with their closely affiliated partners [31], [33], [34], and, when they do respond to unfamiliar calls, they are more likely to do so if the structure of the novel vocalization is similar to those produced by familiar group members [38]. Similarly, Japanese macaques modify the structural characteristics of their calls according to the features of prior vocalizations produced by group members [37]. In addition, the timing of vocal production by individuals participating in affiliative exchanges suggests a system of turn-taking [29], [40]. Finally, infant bushbabies respond vocally to certain types of vocalizations or chains of vocalizations produced by their mothers [41]. When one considers the behaviors of monkeys during vocal exchanges among and between individuals in close proximity of one another, a number of important similarities with human conversation are observed.

Chimpanzees also produce proximal vocalizations directed to conspecific recipients, [24], [26]. Laporte & Zuberbuhler [26] recently reported that female chimpanzees were more likely to produce pant-grunts (a proximal vocalization that is directed to a specific recipient) when encountering a male in the absence of the group’s alpha male, than they were if the alpha male was present. This suggests that the female chimpanzees are able to flexibly deploy these vocal signals depending upon the social context. The increased activity in the left IFG associated with the production of attention-getting calls described above are consistent with this finding, and support the conclusion that at least some vocal signals are produced flexibly by chimpanzees and deployed for specific communicative ends, as has been demonstrated for chimpanzee (and other great ape) manual gestures [4], [5], [6], [8], [9]. The fact that the IFG is involved in the production of (at least some) vocal signals by chimpanzees suggests a level of cortical control that has not been previously identified, and points to marked continuities between the neurobiological structures that support chimpanzee vocal and gestural communication and the neural substrates of human language.

Finally, S2 produced only 2 communicative gestures in the COM condition, and similarly produced 2 gestures in the RES condition. This is particularly interesting, given the pattern of neuronal metabolic activity observed in this subject. It appears that – at least for this subject – the increased activity in the left IFG in the COM condition compared to RES is related exclusively to the production of attention-getting calls, and not manual gestures. It is also worth noting that S4 actually seemed to show increased neuronal metabolic activity in the left IFG in RES compared with COM. It is unclear what accounts for this observation. As described above, the behavior of the chimpanzees during both the COM and RES conditions were not under the immediate control of the experimenter. Notwithstanding, the gestures produced by this subject in the COM condition were not associated with increased activity in the left IFG.

Undoubtedly, manual gestures play an integral role in human language, and most likely played a significant role in its evolution. For example, people produce manual gestures while speaking [42], and gestures actually precede and predict the development of spoken language in young children [43]. However, although it is true that infants use gestures before they produce spoken *words*, they do not use manual communicative gestures before they produce speech *sounds*. In fact, human infants as young as 6 months of age couple consonant-vowel repetitions with rhythmic manual movements [44]. Therefore, this coupling of oro-facial/vocal sounds and manual communicative gestures seen early on in human development is very similar to the communicative behaviors used by captive chimpanzees described above [16].

The data presented in this report suggest that the left IFG was involved in multimodal communicative signaling prior to the split between humans and chimpanzees some 5 million years ago. We propose that over the course of species divergence, humans gained increased control of both the vocal and gestural modalities, and therefore not only achieved unprecedented vocal flexibility, but became more adept at manual tasks such as tool-construction and use. Notwithstanding, these results point to a multimodal origin for human language. Therefore, future work should focus on the concomitant use of both vocal and manual communicative gestures in chimpanzees and other great apes.
